# The significance of changes in platelet concentration during the early phase after severe burn injury in a Chinese mass casualty

**DOI:** 10.1186/s41038-017-0101-0

**Published:** 2017-12-06

**Authors:** Beiming Shou, Junqiang Li, Chenqi Tang, Qian Tan, Dongfeng Zheng, Binwei Sun, Lanjun Nie, Hongwei Zhang, Yanan Jiang, Chunming Wang, Yanwen Wu

**Affiliations:** 1 Department of Burns and Plastic Surgery, The Drum Tower Clinical Medical College, Nanjing Medical University, Nanjing, Jiangsu 210008 China; 2 Department of Burns and Plastic Surgery, Affiliated Drum Tower Hospital, Nanjing University Medical School, Nanjing, Jiangsu 210008 China; 3 Department of Burns and Plastic Surgery, Hospital of Nanjing Fire Service, Nanjing, Jiangsu 210008 China; 4 Department of Burn, Changhai Hospital, The Second Military Medical University, Shanghai, 200433 China; 5 Department of Burn and Plastic Surgery, Affiliated Hospital, Jiangsu University, Zhenjiang, Jiangsu 212001 China; 6 Department of Burn and Plastic Surgery, The Second Affiliated Hospital of Nanjing Medical University, Nanjing, Jiangsu 21000 China; 7 Department of Burns and Plastic Surgery, Hospital of Jiangsu province, Nanjing, Jiangsu 210000 China

**Keywords:** Mass burn casualty, Platelet concentration, Sepsis, MOF

## Abstract

**Background:**

Changes in platelet concentration are common in severe burn patients. Platelets play a key role in the course of disease. This study aims to explore the significance of platelet concentration during the course of the disease in victims of a mass burn casualty.

**Methods:**

A total of 180 patients were involved in the “8.2” Kunshan explosion accident in China. The examined data included age, gender, total burn area (% TBSA), third-degree burn area (% TBSA), and platelet concentration within the first 5 days after the burn injury. The patients were divided into two groups according to four indicators (resuscitation, acute respiratory distress syndrome, acute kidney injury, septic shock). We collected several types of data for the patients and divided the patients into a complication group and non-complication group according to the diagnostic criteria. We analyzed the platelet concentration of the two groups using *t* tests to determine whether significant differences were present. *P* values < 0.05 were considered statistically significant.

**Results:**

The group with successful resuscitation had higher platelet concentration than the failure group on day 3 and day 5. The patients who suffered from acute kidney injury (AKI) and septic shock had a lower platelet concentration than non-sufferers on day 3 and day 5.

**Conclusions:**

The platelet concentration of burn patients can dynamically reflect the pathophysiological changes of the body. It can be used as an early objective indicator of prognosis in mass burn casualty cases.

## Background

Hemostasis disorders are an important element of burn disease pathogenesis, mostly during the acute period [1]. Changes in platelet concentration are common in severe burn patients and are a signal of a severe condition and poor outcome. Because platelet concentration is an early signal of changes in condition, we should attach great importance to the fluctuation of platelet concentration during the treatment process and avoid delaying treatment. Burns are the fourth leading cause of death from unintentional injury in the USA and result in 1.4 million injuries each year. Burn injury is the most severe form of trauma, accounting for ~ 330,000 deaths per year worldwide [2]. “Mass burn casualty” events are those that cause more than 10 burn cases or severe burns in more than five cases. Mass burn casualties have the same cause of injury, similar degrees of injury degree, similar medical treatment times, and high proportions of severe injuries. Because a large number of patients are affected during mass burn casualty events, they are usually sent to several medical centers at the same time. It has been reported that the mortality rate is as high as 97.8% in patients with burns over 70% total body surface area (TBSA) [3]. Crosswise comparisons are beneficial. However, changes in platelet concentration in mass burn casualties are rarely reported. This study collected 180 burn patients’ data from China’s “8.2” Kunshan explosion and analyzed the relationship between platelet concentration and the prognosis of mass burn casualties in hopes of providing some ideas for clinical treatment.

The "8.2" Kunshan explosion was an aluminum dust explosion on August 2, 2014, at a workshop in Kunshan, China, that polished car wheel hubs. A total of 185 people were burned and were sent to 20 medical centers for treatment. The "8.2" Kunshan explosion shared several characteristics with previous burn accidents: The workers experienced an aluminum powder explosion in a closed environment; consequently, their injuries were complex and critical. Most of the patients suffered from not only large areas of severe burns but also inhalation injury caused by heat and the inhalation of hot dust. Kunshan is an economically developed city located in eastern China. It has convenient transportation and is rich in medical resources, which allowed patients to be treated in a timely manner. Health authorities attached great importance to the accident, organizing a large number of relevant medical experts from throughout the country and assembling the necessary goods to support them. All the medical experts launched a multidisciplinary, cooperative treatment effort. Under the government’s organization, labor, material resources, and financial resources were guaranteed, and all patients were well treated.

The purpose of our study was to investigate whether platelet pathology affects the prognosis of mass burn patients. If the platelet concentration reflects pathophysiological changes, it could be used as an early warning indicator. Clinicians could adjust their treatment options and determine the patients’ condition and prognosis during the early stages of injury based on platelet concentration.

## Methods

### Patients

Patient data were collected from victims of the "8.2" Kunshan explosion, which included 180 patients (we excluded five patients whose platelet concentration data were incomplete) from 19 burn centers. The total burn area was 86.83 ± 19.46% TBSA, and the third-degree burn area was 71.92 ± 27.71% TBSA. Patient characteristics are presented in Table 1. The day the casualty occurred was defined as day 1.

**Table 1:** Characteristics of patients

	Total N	%TBSA						Age (years)			
		< 50	50–69	70–89	90–94	95–97	98–100	< 30	30–39	40–49	50–59
Total	180	11	10	26	30	44	59	42	55	77	6
Male	99	7	4	10	18	27	33	32	29	33	5
Female	81	4	6	16	12	17	26	10	26	44	1

*TBSA* total body surface area

### Methods

We observed the platelet concentration of 180 patients within first 5 days of the burn injury and changes in the patients’ conditions within a month. The liquid resuscitation of the patients followed China’s burn formula (1.5 ml/kg/% TBSA). We considered mean arterial pressure greater than 60 mmHg and urinary output of more than 0.5 ml/kg/h successful resuscitation.

Acute respiratory distress syndrome (ARDS) was defined as a P/F ratio ≤ 300; new, bilateral infiltrates on chest X-ray consistent with parenchymal pulmonary disease; and acute onset of disease (within 7 days) that could not be explained by acute left heart failure [4].

Acute kidney injury (AKI) was defined as a urine output < 0.5 mL/kg/h for > 6 consecutive hours and an increase in serum creatinine by ≥ 0.3 mg/mL (≥ 26.5 μmol/L) over 48 h or ≥ 50% over 7 days [5].

The American Burn Association has developed specific guidelines for the diagnosis of sepsis in burn patients. They include higher thresholds for temperature (> 39 °C or < 36.5 °C), HR (> 110 beats/min), and RR (> 25/min) in addition to the presence of thrombocytopenia (platelet concentration < 100,000/ml) and indications of insulin resistance or feeding intolerance [6]. Septic shock was diagnosed according to the Surviving Sepsis campaign of 2012 [7].

The patients were divided into two groups based on four indicators. We collected several types of data for the patients, including temperature, HR, RR, platelet concentration, liquid resuscitation, mean arterial pressure, urinary output, chest X-ray results, and serum creatinine levels. The patients were divided into a complication group and a non-complication group according to the criteria mentioned above. We analyzed the platelet concentration of the two groups using *t* tests to determine whether significant differences existed.

### Statistical analysis

All the data are expressed as the mean ± SD (standard deviation), and the SPSS for Windows 19.0 was used for statistical analysis (SPSS, Inc., Chicago, IL, USA). Statistical analysis was performed using *t* tests for comparisons of different patient exposures on the first, third, and fifth days. The data of all groups obeyed normal distribution (*P* > 0.05). The difference was considered significant when *P* < 0.05.

## Results

### Resuscitation indicators

The patients were divided into two groups: the success group comprised patients who were successfully resuscitated and the failure group comprised all others. We analyzed the platelet concentration on day 1, day 3, and day 5 [Table 2]. There was no significant difference between the two groups on the first day after injury. However, there were statistically significant differences between the two groups on day 3 and day 5. The platelet concentration of the success group was significantly higher than that of the failure group.

**Table 2:** The difference of platelet concentration between successful resuscitation group and failure group

Group	N	Platelet concentration (× 10^9^/L)		
		Day 1	Day 3	Day 5
Success	77	304.44 ± 113.65	77.41 ± 39.98	92.74 ± 43.63
Failure	103	333.5 ± 142.66	60.6 ± 30.61	69.73 ± 28.19
*P* value		> 0.05	< 0.05	< 0.05

### Organ complications

Based on the diagnostic criteria for ARDS, AKI, and septic shock, we divided the patients into two groups and compared the platelet concentration on day 1, day 3, and day 5 [Table 3]. The platelet concentration of the ARDS group and the control group did not show a statistically significant difference. For patients with AKI and septic shock, the platelet concentration on day 3 and day 5 were significantly lower than those of the control group.

**Table 3:** The relationship of the platelet concentration and complications occurrences after severe burn injury

Group	*N*	Platelet concentration (×10^9^/L)		
		Day 1	Day 3	Day 5
ARDS	107	318.79 ± 86.56	67.41 ± 29.98	78.59 ± 22.63
Non-ARDS	73	334.54 ± 104.71	71.96 ± 26.84	85.34 ± 28.21
*P* value		> 0.05	> 0.05	> 0.05
AKI	56	319.18 ± 103.21	65.31 ± 25.66	75.56 ± 33.23
Non-AKI	124	325.83 ± 86.36	76.51 ± 33.43	89.74 ± 43.53
*P* value		> 0.05	< 0.05	< 0.05
Septic shock	129	331.28 ± 102.41	63.2 ± 31.87	77.33 ± 28.41
Non-septic shock	51	328.19 ± 86.36	79.37 ± 33.94	90.56 ± 32.63
*P* value		> 0.05	< 0.05	< 0.05

*ARDS* acute respiratory distress syndrome, *AKI* acute kidney injury

## Discussion

Platelets are derived from the bone marrow megakaryocyte precursor, which contains loose granules and dense granules. Their average life expectancy is approximately 7 to 14 days. Thrombocytes have a variety of biological activities and functions. They are also extremely sensitive inflammatory cells that play an important role in the inflammatory response. Platelets have been preliminarily recognized as able to sense damage to vessel endothelium. Increasing recent evidence suggests that platelets have an indispensable role in regulating inflammatory responses [8–10]. The platelet concentration reflects the production and decay of thrombocytes, which decreases when peripheral blood platelets are destroyed; this decrease also stimulates compensatory hyperplasia of the bone marrow megakaryocytes to produce more platelets [11]. The platelet concentration will increase when the primary disease is under control. Because the platelet concentration can reflect the relationship between the consumption and generation of platelets in the body to some degree, it also shows early changes in conditions. Therefore, to a certain extent, platelet concentration, which can provide an early and sensitive indicator of illness severity and prognosis, also indirectly reflect the body’s ability to respond to disease.

Shock refers to a pathological process characterized by the reduction of the body’s effective circulation blood volume, tissue perfusion deficiency, cellular metabolic disorders, and functional impairment. The essence of burn-related shock is hemorrhagic shock. A major goal of the initial management of burn injuries is to replace extracellular fluid loss in proportion to the % TBSA of the burn. For large burns, intravenous (i.v.) fluid therapy is required to avoid the life-threatening consequences of hypovolemic shock; to this aim, a number of resuscitation formulae have been proposed [12]. Our study found that patients who were successfully resuscitated from shock had a higher platelet concentration level than others. Due to the lack of effective blood volume and hemoglobin, patients who suffer resuscitation failure will experience pathophysiological deterioration and instability of the internal environment. As delayed resuscitation, tissue ischemia, hypoxia reperfusion injury, and acute infection promote the activation of the coagulation system, platelet activation factor (PAF) is released, resulting in further decreases in the number of platelets.

Sepsis and multiple organ dysfunction syndrome (MODS) caused by sepsis are the principal reasons for death in burn patients [13]. Several studies have shown variation in the rates, mortality, and characteristics of patients with MODS according to the type of patients, sample size, definition, and involved organs. In many burn centers, MODS is still a leading cause of death in patients with severe burns [14]. Due to the strong and persistent stress stimulation after severe burns combined with platelet adhesion and aggregation, active substances such as PAF, prostaglandin G_2_ (PGG_2_), prostaglandin H_2_ (PGH_2_), and thromboxane A_2_ (TXA_2_), which are released by platelets, produce complex biological effects. These active substances, which can strengthen the inflammatory response, affect the regulation of blood flow, change vasodilator levels and permeability, and alter the coagulation state, resulting in the contraction of blood vessels, the aggregation of inflammatory cells, and the release of a large number of inflammatory mediators. To a certain extent, these changes can endanger the survival of cells and lead to organ failure. As a result, the excessive production of platelets is a sign of poor prognosis. PAF was directly involved in shock, sepsis, and organ failure. Changes in peripheral platelet concentration indicate that the rapid decline in platelet concentration is related to multiple organ failure (MOF).

Sepsis refers to systemic inflammatory response syndrome (SIRS) caused by infection, which is a serious complication of shock, burns, trauma, infection, and major surgery. In sepsis, abnormal platelet activation and neutrophil paralysis is well recognized. Platelet activity is characterized by the contribution to disseminated intravascular coagulation (DIC) and the enhanced inflammation response [15]. Severe burns complicated with sepsis are an important cause of MODS [16]. Most organ damage occurs during the first week post-burn; additional damage may occur within 4 weeks post-burn, but the rate of organ damage slows in the third week. The pathogenesis of sepsis is complex and has not yet been fully elucidated; however, it includes infection, inflammation, tissue damage, and dysfunctions of the immune system, blood coagulation, metabolism, neuroendocrine system, and immunologic regulating network. Thrombocytopenia is one characteristic of sepsis that increases the risk of death [17]. Research proves that thrombocytopenia is associated with sepsis, prolonged hospital stays, and increased mortality [18]. The present study found statistically significant differences between patients with septic shock and those with non-septic shock on day 3 and day 5, yet there were no significant differences between these patients on the day of injury. This difference suggests that platelet concentration is an early indicator of septic shock. As an indicator of diffuse intravascular coagulation, thrombocytopenia is related to inflammatory mediators [19]. Treating the primary disease and providing anti-infective therapy are more effective than simply correcting the platelet concentration. A downward trend in platelet concentration is more informative than whether the platelet concentration is within the normal range. This report shows that platelet concentration is associated with reductions in the mortality rate and that the degree of decrease in the platelet concentration is positively correlated with the severity of sepsis [20]. Consequently, observing the daily changes in platelet concentration and considering them as a sign of the progress of anti-inflammatory treatment offers important guiding significance.

The previous analysis shows that platelet concentration can provide an objective and sensitive early indicator of severe burn patients’ condition and outcome. The reasons are as follows: A platelet’s life cycle is short and very few platelets are stored in the marrow, because the platelet concentration changes during the early stages of disease, which can sensitively show the severity of a patient’s condition. Platelets play an important role in the inflammatory response; therefore, the degree of the inflammatory response is associated with the platelet concentration. Disease progression is associated with the risk of reducing the platelet concentration, and reduced platelet concentration may also lead to the exacerbation of disease. They are connected and influence each other.

## Conclusions

Our study confirms the prognostic role of platelet concentration in pathophysiological changes. Platelet concentration is an early warning indicator for the early treatment of mass burn casualties that can dynamically reflect pathophysiological changes in body. It is also a common and repeatable clinical indicator. As platelet concentration changes occur before the appearance of clinical symptoms and can be easily determined, clinicians should pay increased attention to platelet changes to inform adjustments of therapy. Additionally, platelet concentration can be used as an early objective indicator of prognosis in the case of mass burn casualties.

## Abbreviations

AKI: Acute kidney injury
ARDS: Acute respiratory distress syndrome
DIC: Disseminated intravascular coagulation
MODS: Multiple organ dysfunction syndrome
MOF: Multiple organ failure
PAF: Platelet activation factor
PGG_2_: Prostaglandin G_2_
PGH_2_: Prostaglandin H_2_
SIRS: Systemic inflammatory response syndrome
TBSA: Total body surface area
TXA_2_: Thromboxane A_2_

## References

[1] Kowal-Vern A, Sharp-Pucci MM, Walenga JM, Dries DJ, Gamelli RL (1998). Trauma and thermal injury: comparison of hemostatic and cytokine changes in the acute phase of injury. Trauma.

[2] Peden M, McGee K, Sharma G (2002). The injury chart book: a graphical overview of the global burden of injuries.

[3] Kraft R, Herndon DN, Almousawi AM, Williams FN, Finnerty CC, Jeschke MG (2012). Burn size and survival probability in paediatric patients in modern burn care: a prospective observational cohort study. Lancet.

[4] Bordes J, Lacroix G, Esnault P, Goutorbr P, Cotte J, Dantzer E (2014). Comparison of the Berlin definition with the American European Consensus definition for acute respiratory distress syndrome in burn patients. Burns.

[5] McIlroy DR, Argenziano M, Fankas D, Umann T, Sladen RN (2013). Incorporating Oliguria into the diagnostic criteria for acute kidney injury after on-pump cardiac surgery: impact on incidence and outcomes. J Cardiothorac Vasc Anesth.

[6] Greenhalgh DG, Saffle JR, Holmes JH, Gamelli RL, Palmieri TL, Horton JW (2007). American burn association consensus conference to define sepsis and infection in burns. J Burn Care Res.

[7] D Annane, E Bellissant, J-M Cavaillon. Septic shock. Lancet 2005; 365(9451):363-365. 10.1016/S0140-6736(04)17667-8 15639681

[8] Semple JW, Italiano JE, Freedman J (2011). Platelets and the immune continuum. Nat Rev Immunol.

[9] Asaduzzaman M, Lavasani S, Rahman M, Zhang S, Braun OO, Jeppsson B (2009). Platelets support pulmonary recruitment of neutrophils in abdominal sepsis. Crit Care Med.

[10] Li Z, Yang F, Dunn S, Gross AK, Smyth SS (2011). Platelets as immune mediators: their role in host defense responsesand sepsis. Thromb Res.

[11] Pavic M, Milevoj L (2007). Platelet count monitoring in burn patients. Biochemica Medica.

[12] Dulhunty JM, Boots RJ, Rudd MJ, Muller MJ, Lipman J (2008). Increased fluid resuscitation can lead to adverse outcomes in major-burn injured patients, but low mortality is achievable. Burns.

[13] Xu C, Fan K, Xie L, Chen W, Wang L (2014). Evaluation of optimized continuous venovenous hemodiafiltration therapy efficiency in severe burn patients with sepsis. Burn Trauma.

[14] Nguyen LN, Nguyen TG (2009). Characteristics and outcomes of multiple organ dysfunction syndrome among severe-burn patients. Burns.

[15] Wang XU, Qin W, Sun B (2014). New strategy for sepsis: targeting a key role of platelet-neutrophil interaction. Burn Trauma.

[16] Chen J, Yan H, Luo G, Luo Q, Li X, Zhang J (2013). Characteristics of burn deaths from 2003 to 2009 in a burn center: a retrospective study. Burn Trauma.

[17] Marck RE, Montagne HL, Tuinebreijer WE, Breederveld RS (2013). Time course of thrombocytes in burn patients and its predictive value for outcome. Burns.

[18] Wu Q, Ren J, Wu X, Wang G, Gu G, Liu S (2014). Recombinant human thrombopoietin improves platelet counts and reduces platelet transfusion possibility among patients with severe sepsis and thrombocytopenia: a prospective study. Burns.

[19] Levi M (2005). Platelet in sepsis. Hematology.

[20] Vandijick DM, Blot SI, De Waele JJ, Hoste EA, Vandewoude KH, Decruyenaere JM (2010). Thrombocytopenia and outcome in critically ill patients with bloodstream infection. Heart Lung.

